# Supplementary material for: Estimating the Cholesterol Affinity of Integral Membrane Proteins from Experimental Data
Source: Biochemistry. 2023 Dec 15;63(1):19–26. doi: 10.1021/acs.biochem.3c00567 (PMC10765374; doi:10.1021/acs.biochem.3c00567)
Supplement: Supplementary file 1 — bi3c00567_si_001.pdf [file bi3c00567_si_001.pdf]

Supporting Information

A METHOD FOR ESTIMATING THE CHOLESTEROL AFFINITY OF INTEGRAL MEMBRANE PROTEINS FROM EXPERIMENTAL DATA

Theodore L. Steck[#], S. M. Ali Tabei[§] and Yvonne Lange*

[#]Department of Biochemistry and Molecular Biology, University of Chicago, Chicago, Il 60637

[§]Department of Physics, University of Northern Iowa, Cedar Falls, Iowa 50614

[*]Department of Pathology, Rush University Medical Center, Chicago, Il 60612

Table of contents:

1. The model
2. Computational code
3. Derivation of text Eq. (6)
4. References

S1. The Model (reproduced from the text)

This model simulates the binding of sterol molecules to a ligand within a phospholipid bilayer and estimates the affinity and stoichiometry of the reaction. It resembles an earlier model that treats the binding of water-soluble ligands such as cytolysins to membrane sterols.[1] In contrast, the present version places the ligand in the same membrane compartment as the sterol and the phospholipid. We assume that a ligand (L) associates with cholesterol (C) in competition with the membrane phospholipids (P). The important parameters are therefore the sterol association constants and stoichiometries for the phospholipids and the ligand. The compartment size is $\alpha = (C_T + P_T + L_T)$ where the subscript T denotes total. The chemical activities of the reactants are ideal and expressed as their concentrations in mole fractions.

These are C_f/α, P_f/α and L_f/α, where f denotes the free form of the reactant. CP_r denotes lipid complexes and C_nL denotes cholesterol-ligand (namely, protein) complexes.

We postulate an association equilibrium for the sterol and phospholipid:

$$C_f + r\ P_f \leftrightarrow CP_r \tag{1}$$

The dimensionless association constant for complexation is then

$$K_P = [(CP_r)/\alpha] / \{[(C_f)/\alpha] \times [(P_f)/\alpha]^r\} = (CP_r) \times \alpha^r / [(C_f) \times (P_f)^r] \tag{2}$$

Many integral membrane proteins such as the transporters considered here are oligomeric.[2, 3] For simplicity, we assume that the ligand (L) is an oligomer composed of identical subunits that bind n sterol molecules in a fully cooperative all-or-none fashion; a concerted rather than sequential reaction.[4] Hence,

$$n\ C_f + L_f \leftrightarrow C_nL \tag{3}$$

Designating the dimensionless association constant for the formation of an n-mer of L as K_{Ln}, we have

$$K_{Ln} = [(C_nL)/\alpha] / \{[(C_f)/\alpha]^n \times (L_f)/\alpha\} = (C_nL) \times \alpha^n / [(C_f)^n \times (L_f)] \tag{4}$$

S2. Computational code

This is a pseudocode for the model (version of July 11, 2023). It describes the equilibrium binding of an oligomeric ligand (L) to cholesterol (C) in competition with one or two membrane phospholipids, A and B. The original MATLAB code is available upon request.

n sets the number of monomers in the oligomer, CnL.

r sets the cholesterol:phospholipid stoichiometry of phospholipid A, CA_r where r = 1 or 2.

s sets the cholesterol:phospholipid stoichiometry of phospholipid B, CB_s where s = 1 or 2.

KA sets the sterol association constant for phospholipid A.

KB sets the sterol association constant for phospholipid B.

KL sets the association constant for cholesterol binding to one subunit of the oligomeric ligand.

PT = 1 sets the total moles phospholipid to unity.

CT is moles total cholesterol, the independent variable.

AT and BT set the proportions of phospholipids A and B where AT + BT = 1.

LT sets the total moles of the ligand. Typically, 1E-3 or 1E-5.

Cf is moles uncomplexed (free) cholesterol.

```
AT=x*PT; BT=1-AT; %%% x sets the fraction of PT in lipid A and therefore in lipid B.

CT=(1E-3:0.008:1.5); this sets the range of total cholesterol.

alpha=CT+PT+LT; %% this is the total moles in the system.

length_C=length(CT);

y0=[eps,eps,eps,eps];

for i=1:length_C
  %MATLAB nonlinear numerical solver
  C=fsolve(@(y) complexC(y,KA,KB,KL,AT,BT,LT,n,r,s,CT(i),alpha(i)),y0);
  CAr(i)=C(1);
  CBs(i)=C(2);
  CnL(i)=C(3);
  y0=C+eps;

end

Cf=CT-CAr-CBs-n*CnL; %% this is the amount of free cholesterol.
G=CnL./LT; %% this is the fraction of LT bearing C as a f(CT).
H=1-CnL./LT; %% this is the fractional inhibition by the binding of C  to L as a f(CT).
J=CT./alpha; %% this is the mole fraction of total cholesterol.
Z=Cf/(CT+PT+LT); this is the mole fraction of free cholesterol.

figure (1) %%this plots the fraction of L bearing C vs. the mole fraction total cholesterol.
plot(J,G,'s')
xlabel({'mf CT'});
ylabel('CnL/LT');

figure (2) %% this plots the fraction of free C vs. mole fraction total cholesterol.
plot(J,Cf,'s')
xlabel({'mf CT'});
ylabel('Cf/CT');

figure (3) %% this plots the fraction of L inhibited by C vs. mole fraction total cholesterol.
plot(J,H,'s')
xlabel({'mf Ct'});
```

```
ylabel('1-CnL/LT');

T = array2table([J',CT',Cf',alpha',G',H']);
T.Properties.VariableNames={'mf_CT','CT','Cf','alpha','CnL','1-G'};
writetable(T,'file name.xlsx'); exportable table of results

%Defining the model
function f = complexC(y,KA,KB,KL,AT,BT,LT,n,r,s,CT,alpha)
 f(1)=alpha.^r.*y(1)-KA*(CT-y(1)-y(2)-n*y(3)).*(AT-r*y(1)).^r;
 f(2)=alpha.^s.*y(2)-KB*(CT-y(1)-y(2)-n*y(3)).*(BT-s*y(2)).^s;
 f(3)=alpha.^n.*y(3)-KL*(LT-y(3)).*(CT-y(1)-y(2)-n*y(3)).^n;

end
```

S3. Derivation of equations for the binding of cholesterol to ligands at half-saturation

S3.a. The model posits that the protein is a homooligomer that binds cholesterol with perfect cooperativity. Text Eq. 4 (reproduced in Section S.1) shows that at half-saturation of the protein—that is, when $(C_nL) = (L_f)$, the association constant for an n-mer is equal to that of one of its identical subunits raised to the nth power; that is, $K_{Ln} = (K_{L1})^n$. It also follows from text Eq. 4 that, at half-saturation, the free cholesterol concentration in the binding reaction is given by

$$(C_f)_{1/2} = \alpha / (K_{Ln})^{1/n} = \alpha / K_{L1} \qquad \text{(S1)}$$

Therefore, the half saturation point of a protein, $(C_f)_{1/2,}$ is independent of the sterol binding stoichiometry, n, and the cholesterol-dependent isotherms of the protein will all intersect at $(C_f)_{1/2}$ (not shown). Eq. (S1) is reproduced as Eq. (5) in the text.

S3.b. Figures 3 to 6 show that isotherms for a protein with varied n values intersect close to the half saturation point of the total cholesterol concentration, $(C_T)_{1/2}$. That the $(C_T)_{1/2}$ for a protein is essentially independent of n is shown below.

By definition,

$$(C_T) = (C_f) + (CP_r) + n \times (C_nL) \qquad \text{(S2)}$$

Text Eq. (2) (reproduced in Section S.1) states that

$$K_P = (CP_r) \times \alpha^r / [(C_f) \times (P_f)^r]$$

Substituting for CP_r from Eq. (S2) yields,

$$K_P = [(C_T) - (C_f) - n \times (C_nL)] \times \alpha^r / [(C_f) \times (P_f)^r] \quad \text{(S3)}$$

Rearranging Eq. (S3) and raising it to the nth power yields

$$(C_f)^n = [(C_T) - (C_f) - n \times (C_nL)]^n \times (\alpha^{r+n}) / [K_P^n \times (P_f)^{r+n}] \quad \text{(S4)}$$

From text Eq. (4),

$$K_{Ln} = (C_nL) \times \alpha^n / [(C_f)^n \times (L_f)]$$

Substituting Eq. (S4) into this equation yields

$$K_{Ln} = (C_nL) \times \alpha^n \times K_P^n \times (P_f)^{r+n} / \{(L_f) \times \alpha^{r+n} \times [(C_T) – (C_f) – n \times (C_nL)]^n\} \quad \text{(S5)}$$

At half saturation, $(C_nL)_{1/2} = (L_f)_{1/2}$

So,

$$K_{Ln} = \alpha^n \times K_P^n \times (P_f)_{1/2}^{r+n} / \{\alpha^{r+n} \times [(C_T)_{1/2} – (C_f)_{1/2} – n \times (C_nL)_{1/2}]^n\} \quad \text{(S6)}$$

It follows that

$$[(C_T)_{1/2} - (C_f)_{1/2} - n \times (C_nL)_{1/2}]^n = \alpha^n \times (K_P)^n \times (P_f)_{1/2}^{r+n} / [\alpha^{r+n} \times K_{Ln}] \quad \text{(S7)}$$

Taking the nth root of both sides yields

$$(C_T)_{1/2} - (C_f)_{1/2} - n \times (C_nL)_{1/2} = \alpha \times K_P \times (P_f)_{1/2}^r / [\alpha^r \times (K_{Ln})^{1/n}] \quad \text{(S8)}$$

At half saturation, $(K_{Ln})^{1/n} = K_{L1}$ and $(C_f)_{1/2} = \alpha/K_{L1}$. Therefore,

$$(C_T)_{1/2} - \alpha/K_{L1} - n \times (C_nL)_{1/2} = \alpha \times K_P \times (P_f)_{1/2}^r /[\alpha^r \times K_{L1}] \quad \text{(S9)}$$

Therefore,

$$(C_T)_{1/2} - n \times (C_nL)_{1/2} = [\alpha/K_{L1}] \times \{1 + [K_P \times (P_f)_{1/2}{}^r / \alpha^r]\} \qquad \text{(S10)}$$

Because n x (CnL) << (C_T), Eq. (S10) is well approximated as

$$(C_T)_{1/2} \approx [\alpha/K_{L1}] \times \{1 + [K_P \times (P_f)_{1/2}{}^r / \alpha^r]\} \qquad \text{(S11)}$$

Therefore, as can be seen in Figures 3-6, the half-saturation point observed for the isotherms of a protein with affinity K_{Ln} is a function of K_{L1} and K_P but is essentially independent of n. Simulations show that this approximation is good to within 1%. Eq. (S11) is presented in the text as Eq. (6).

References

[1] Lange, Y., Tabei, S. M. A., and Steck, T. L. (2023) A basic model for the association of ligands with membrane cholesterol: application to cytolysin binding, *Journal of Lipid Research 64*, 100344.

[2] Yifrach, O., Zandany, N., and Shem-Ad, T. (2009) EXAMINING COOPERATIVE GATING PHENOMENA IN VOLTAGE-DEPENDENT POTASSIUM CHANNELS: TAKING THE ENERGETIC APPROACH, In *Methods in Enzymology, Vol 466: Biothermodynamics, Pt B* (Johnson, M. L., Holt, J. M., and Ackers, G. K., Eds.), pp 179-+, Elsevier Academic Press Inc, San Diego.

[3] Banerjee, K., Das, B., and Gangopadhyay, G. (2013) On the estimation of cooperativity in ion channel kinetics: Activation free energy and kinetic mechanism of Shaker K+ channel, *The Journal of Chemical Physics 138*, 165102.

[4] Morea, V., Angelucci, F., Tame, J. R. H., Di Cera, E., and Bellelli, A. (2022) Structural Basis of Sequential and Concerted Cooperativity, *Biomolecules 12*, 1651.
